# Epidemiology and Antifungal Susceptibility of Candida Bloodstream Isolates From a Tertiary Care Center in Central India

**DOI:** 10.7759/cureus.97120

**Published:** 2025-11-17

**Authors:** Arati A Bhadade, Gajendra Singh, Tadepalli Karuna, Madhu Panthi, Ankita Beniwal, Anand K Maurya, Neha Upadhyay, V. R Reddy, Apoorva Devgade, Sagar Khadanga

**Affiliations:** 1 Microbiology, All India Institute of Medical Sciences, Bhopal, Bhopal, IND; 2 General Medicine, All India Institute of Medical Sciences, Bhopal, Bhopal, IND

**Keywords:** antifungal resistance, antifungal susceptibility testing, bloodstream infection (bsi), candida species, non-albicans candida

## Abstract

Background: Fungal bloodstream infections (BSIs), particularly candidemia, are recognized as major contributors to hospital-acquired infections and mortality. Early species and antifungal drug resistance pattern identification are crucial for timely intervention and improved outcomes. Consequently, this study aimed to examine the distribution of *Candida* species isolated from blood cultures and assess their antifungal susceptibility profiles.

Methods: This retrospective observational study was conducted between January and December 2024. *Candida* species were identified via Matrix-Assisted Laser Desorption/Ionization Time-of-Flight Mass Spectrometry (MALDI TOF-MS), and antifungal susceptibility testing was performed using the Sensititre YeastOne method.

Results: Candidemia was detected in 118 (0.73%) of total blood culture samples (*N* = 16,040). *Candida tropicalis* was the most frequently isolated species (38, 32.20%), followed by *Candida albicans* (17, 14.41%) and *Candida parapsilosis* (16, 13.56%). Additionally, *C. tropicalis* exhibited the highest resistance to azoles, particularly posaconazole (30, 78.95%). In *C. albicans*, the highest resistance was observed against posaconazole (3, 17.65%) and less with other azoles. Similarly, *C. parapsilosis* demonstrated greater resistance to fluconazole (4, 25%). *Nakaseomyces glabrata* displayed high resistance to voriconazole (11, 84.62%) and posaconazole (8, 61.54%). Furthermore, *Candida auris *isolatesexhibited greaterresistance to fluconazole (7, 87.5%) and caspofungin (4, 50%).

Conclusions: The increasing prevalence of non-albicans Candida (NAC) species and the rising resistance to commonly used antifungals, particularly azoles, underscore the importance of timely diagnosis, species-level identification, antifungal drug resistance testing, and targeted antifungal therapy for candidemia. Higher antifungal drug resistance to posaconazole and fluconazole across several Candida isolates highlights the importance of routine antifungal drug resistance testing for Candida isolates in BSIs. Echinocandins are generally effective against most *Candida* species; however, they should be used cautiously in patients with *C. auris* BSI, as *C. auris* isolates may exhibit multidrug resistance, including resistance to echinocandins.

## Introduction

Bloodstream infections (BSI) due to *Candida* species are the leading cause of invasive fungal infections worldwide and are associated with high morbidity and mortality [1]. Candidemia is the fourth most common cause of nosocomial BSIs globally [1]. According to reports from the Centers for Disease Control and Prevention (CDC), approximately one-third of patients with candidemia die during hospitalization (crude mortality rate) [2]. In the United States alone, an estimated 25,000 cases of candidemia occur annually [3]. Similarly, the prevalence of candidemia in India ranges from 1.31%-3.41% [4,5]. Candidemia is a major public health concern owing to the poor patient outcomes, prolonged hospital stays, increased healthcare costs, and an attributable mortality rate that varies from 5% to 58% [6,7].

Known risk factors for candidemia include immunosuppressive therapies (e.g., chemotherapy), exposure to broad-spectrum antibacterial agents, prolonged use of central venous catheters, undergoing mechanical ventilation, administration of total parenteral nutrition, and hemodialysis [8]. Early diagnosis and treatment of candidemia are challenging due to the lack of specific clinical manifestations. Increasing use of broad-spectrum antibiotics, an ever-expanding range of immunosuppressive disease states (e.g., HIV/AIDS) and treatments (e.g., for cancer and following solid organ transplantation), as well as advances in intensive care medicine, have led to the rising incidence of invasive candidiasis, particularly candidemia, over the past two decades [9].

The epidemiology of species causing candidemia is ever-changing. Although C. albicans was considered the most common species responsible for candidemia in the last two decades, currently, BSI caused by non-albicans *Candida* (NAC) species, such as *Candida tropicalis*, *Nakaseomyces glabrata* (formerly *C. glabrata*), and *Candida parapsilosis*, dominate the epidemiology [10]. In addition, *Candida auris*, which is a major emerging global pathogen, has been increasingly isolated from candidemia cases. Moreover, it is especially contributing to increased mortality caused by late diagnosis, owing to poor identification by conventional methods and multidrug-resistant strains [11]. Several other unusual species, including *Meyerozyma guilliermondii* (*C. guilliermondii*), *Clavispora lusitaniae* (*C. lusitaniae*), and *Kluveromyces marxianus* (*C. kefyr*), have recently been isolated from patients with candidemia, thereby posing a new threat to hospitalized patients. This change in epidemiology has been associated with the increased use of antifungal agents for prophylactic and empirical treatments, whereby several species were found to be less susceptible to azoles. This has resulted in the propagation of costlier alternatives (e.g., echinocandins) for the treatment of candidemia caused by NAC [10]. Therefore, early and accurate identification of *Candida* species in patients with BSI is important for optimizing the treatment by selecting the correct first-line antifungal drugs, and ultimately reducing morbidity as well as mortality.

The emergence of antifungal resistance remains an ever-present threat due to the limited number of antifungal agents, with no significant potential new drugs in the pipeline [11]. Intrinsic and acquired resistance to various antifungals is becoming a major problem in the treatment of candidemia [11]. Although fluconazole is the most commonly used antifungal treatment for this disease, approximately 6% of all *Candida* bloodstream isolates tested at CDC are resistant to fluconazole [2]. Moreover, an increasing number of *Candida* species that are resistant to first-line antifungal treatments (e.g., azoles or echinocandins) are being identified, particularly in high antifungal use settings, where almost all current treatment options are being eliminated [12]. This trend is paralleled by the increased clinical prevalence of multidrug-resistant isolates (e.g., azole and echinocandin-resistant *N. glabrata* and multidrug-resistant *C. auris*). *C. auris* is usually intrinsically resistant to fluconazole (93%), with varying resistance to the echinocandins (7%) and polyenes (35%) [13]. Additionally, 41% of *C. auris* isolates are reported as multidrug-resistant [13] as well, and 4% of strains are pan-drug-resistant to azoles, polyenes, and echinocandins [13]. The increasing clinical prevalence of multidrug-resistant *Candida* species such as *N. glabrata* and *C. auris* highlights the potential for these fungi to pose a serious future threat. Therefore, it is crucial to identify the *Candida* species causing sepsis and determine their antifungal susceptibility pattern to select the right drug that will provide the appropriate coverage against them.

At present, very little data are available for candidemia profiles and antifungal drug resistance patterns in India, particularly in the central region. Answering the existing knowledge gaps and defining a personalized approach to the diagnosis and treatment of candidemia is of paramount importance. Consequently, this study investigated the species distribution and antifungal drug resistance pattern of *Candida* species isolated from blood culture samples in a tertiary care institute, to improve the prognostic outcomes associated with them.

## Materials and methods

This retrospective observational study was conducted in the Department of Microbiology at the tertiary care center in central India. The study was conducted over one year, from January 1, 2024, to December 31, 2024. All *Candida* isolates that were grown in blood culture samples received from patients clinically suspected of sepsis were screened for inclusion in the study.

Inclusion criteria

The *Candida* isolates obtained from the blood culture samples were included only if both the following criteria were fulfilled:

(1) Identification of *Candida* species was confirmed using the Matrix-Assisted Laser Desorption/Ionization Time-of-Flight Mass Spectrometry (MALDI TOF-MS; Bruker Biotyper, Bruker Daltonics GmbH, Bremen, Germany) technique.

(2) *Candida* isolates were tested for antifungal susceptibility by using the Sensititre YeastOne method.

Exclusion criteria

Duplicate isolates were obtained from the blood culture samples of the same patients.

Antifungal susceptibility testing (AFST)

AFST was performed using the Sensititre YeastOne method, as per the manufacturer’s instructions. *Candida* *paropsilosis* ATCC 22019 strain was used as the QC strain, and all results were within the recommended range. The results were interpreted based on CLSI M60 guidelines (2nd edition, 2020) and available epidemiological cutoff values (ECVs), for which the breakpoints are not available [14,15]. A total of nine antifungal agents were tested, including azoles (fluconazole, voriconazole, posaconazole, and itraconazole), polyene (amphotericin B), echinocandins (caspofungin, anidulafungin, and micafungin), and 5-flucytosine.

Data analysis

The frequencies and percentages for different categorical variables were calculated. The Fisher's exact test and chi-square test were used to calculate *P*-values for assessing the significance of associations between variables. For the rare *Candida* isolates whose breakpoints are not available, the geometric mean of minimum inhibitory concentrations (MICs) was calculated for analysis. All analyses were performed using the GraphPad Prism program Version 8.4.2 for Windows (GraphPad Software, San Diego, CA). A *P*-value < 0.05 was considered statistically significant.

## Results

**Figure 1:**
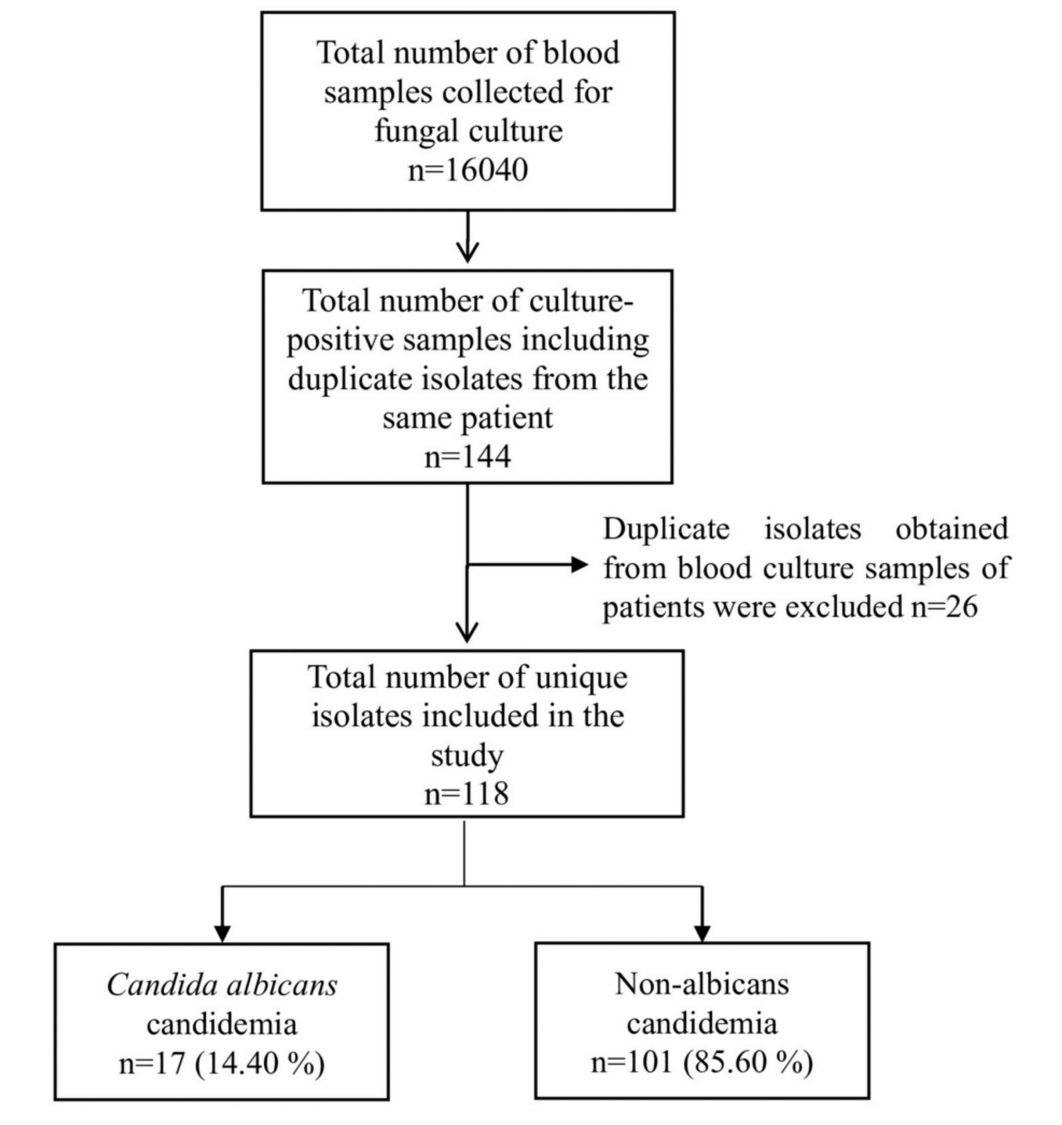
Workflow for the inclusion of fungal blood culture isolates in the study. Image credit: Gajendra Singh.

This retrospective study investigated the prevalence, demographic factors, and antifungal susceptibility patterns of candidemia in patients admitted to a tertiary care hospital. Figure 1 illustrates the workflow for the inclusion of blood culture isolates in the study. A total of 16,040 blood culture samples from patients with suspected sepsis were received during the one-year study period, out of which 144 were found to be culture-positive for *Candida* species. Of these 144 culture-positive samples, only 118 (0.73%) unique isolates were included in the final analysis. In our study, candidemia caused by NAC species (101, 85.60%) was observed to be more common than that caused by *C. albicans* (17, 14.40%).

The clinical and demographic data of patients affected with NAC and *C. albicans* candidemia cases are summarized in Table 1. NAC candidemia was more common in the age group of 19-60 years (54, 53.4%), whereas *C. albicans* candidemia was more common in the 0-18 years age group (8, 47%). Overall, candidemia cases were higher in males (68, 57.63%) than in females (50, 42.37%). Among all the cases, 82 (69.5%) were reported from patients admitted to general wards, whereas 36 (30.5%) were from those in the intensive care units (ICUs).

**Table 1:** Clinical and demographic data of patients with candidemia caused by non-albicans Candida (NAC) species and Candida albicans. Values are expressed as number (*n*), percentage (%). ICU, intensive care unit

Factor	Isolated candida species (*n *= 118), *n* (%)	Non-albicans candidemia (*n *= 101), *n* (%)	*Candida albicans* candidemia (*n *= 17), *n* (%)	Fisher's exact test/chi-square	*P*-value
Age (Year)					
0-18	42 (35.59)	34 (33.67)	8 (47.06)	3.56	0.168
19-60	59 (50)	54 (53.46)	5 (29.42)		
>60	17 (14.41)	13 (12.87)	4 (23.52)		
Gender					
Female	50 (42.37)	42 (41.58)	8 (47.05)	0.1786	0.6726
Male	68 (57.63)	59 (58.42)	9 (52.95)		
Patient location					
Wards	82 (69.5)	71 (70.29)	11 (64.70)	0.2146	0.6432
ICU	36 (30.5)	30 (29.71)	6 (35.30)		

**Figure 2:**
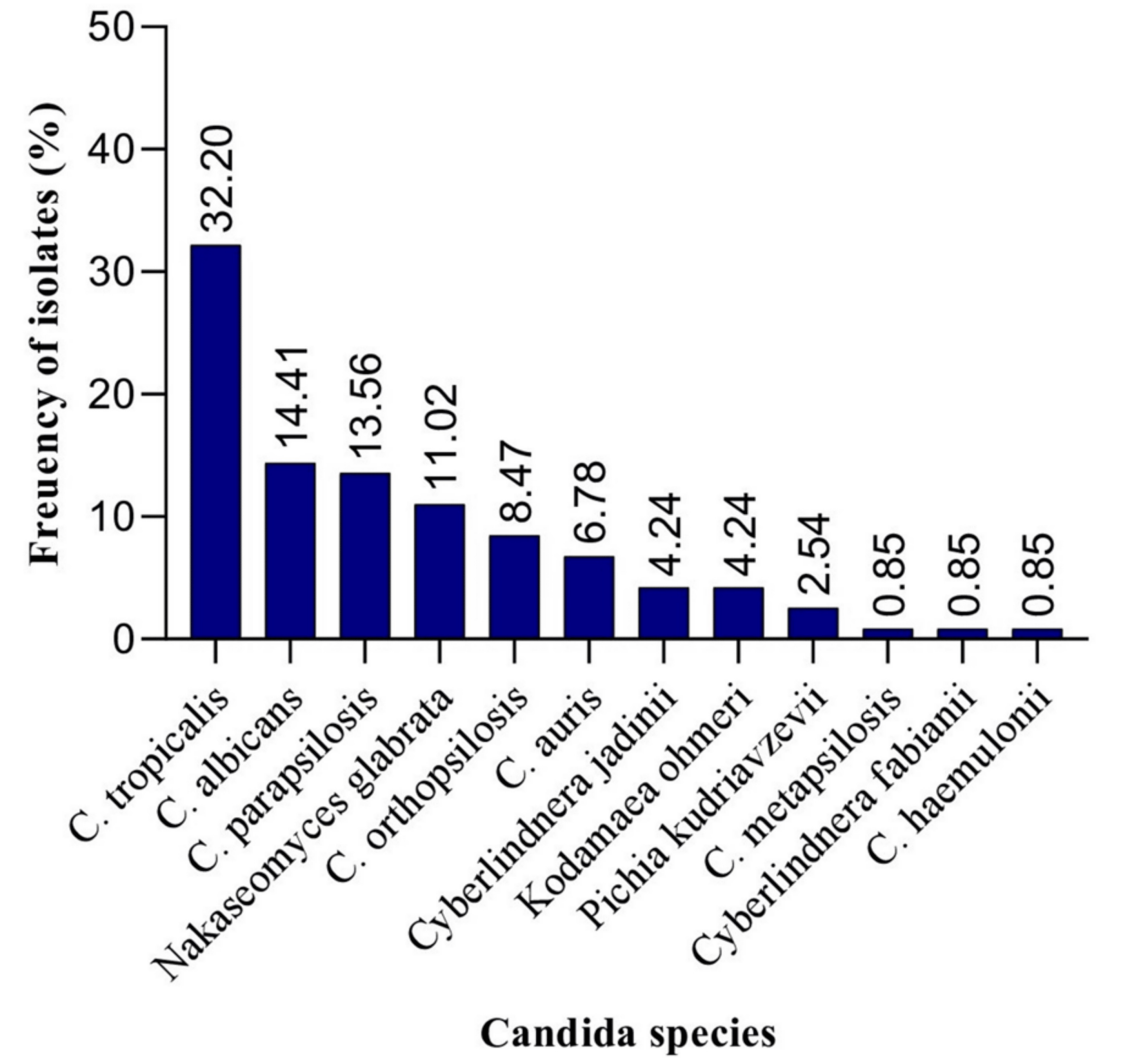
Distribution of Candida species isolated from candidemia isolates (n = 118).

Figure 2 depicts the distribution of various *Candida* species isolated from blood. *C. tropicalis* was the most frequently isolated species (38, 32.20%) of the cases, followed by *C. albicans* (17, 14.41%), *C. parapsilosis* (16, 13.56%), *N. glabrata* (13, 11.02%), *C. orthopsilosis* (10, 8.47%), *C. auris* (8, 6.78%), and *Pichia kudriavzevii* (3, 2.54%). Other less common species included *Cyberlindnera jadinii* (5, 4.24%) and *Kodamaea ohmeri* (5, 4.24%), whereas *C. metapsilosis*, *Cyberlindnera fabianii*, and *Candida haemulonii* were identified in 1 (0.85%) isolate during the study period.

The antifungal resistance patterns of commonly isolated *Candida* species are summarized in detail in Table 2. *C. tropicalis* exhibited the highest resistance to posaconazole (30, 78.95%), followed by voriconazole and fluconazole (7, 18.42%, for both). Additionally, lower resistance rates were noted for itraconazole (5, 13.16%), anidulafungin (5, 13.16%), micafungin (3, 7.89%), caspofungin (2, 5.26%), and amphotericin B (1, 2.63%). Among *C. albicans*, the highest resistance was against posaconazole (3, 17.65%), with high susceptibility to fluconazole and itraconazole (only one resistant isolate each).

**Table 2:** Antifungal resistance patterns of commonly isolated Candida species (n = 105). Values are expressed as number (*n*) and percentage. S, susceptibility; SDD, susceptible-dose dependent; I, intermediate; R, resistance

Antifungal medication	Name of the organisms						
	*Candida tropicalis* (*n* = 38), *n* (%)	*Candida albicans *(*n* = 17), *n* (%)	*Candida parapsilosis* (*n* = 16), *n* (%)	*Nakaseomyces glabrata *(*n* = 13), *n* (%)	*Candida orthopsilosis* (*n* = 10), *n* (%)	*Candida auris *(*n* = 8), *n* (%)	*Pichia kudriavzevii* (*n* = 3), *n* (%)
Fluconazole							
S	25 (65.79)	16 (94.12)	12 (75.00)	0 (0.00)	9 (90.00)	1 (12.5)	0 (0.00)
SDD	6 (15.79)	0 (0.00)	0 (0.00)	10 (76.92)	0 (0.00)	0 (0.00)	0 (0.00)
I	0 (0.00)	0 (0.00)	0 (0.00)	0 (0.00)	0 (0.00)	0 (0.00)	2 (66.67)
R	7 (18.42)	1 (5.88)	4 (25.00)	3 (23.08)	1 (10.00)	7 (87.5)	1 (33.33)
Voriconazole							
S	12 (31.58)	15 (88.24)	12 (75.00)	2 (15.38)	9 (90.00)	-	3 (100.00)
SDD	-	-	-	-	-	-	-
I	19 (50.00)	2 (11.76)	4 (25.00)	0 (0.00)	0 (0.00)	-	0 (0.00)
R	7 (18.42)	0 (0.00)	0 (0.00)	11 (84.62)	1 (10.00)	-	0 (0.00)
Posaconazole							
S	8 (21.05)	14 (82.35)	16 (100.0)	5 (38.46)	10 (100.0)	-	2 (66.67)
SDD	-	-	-	-	-	-	-
I	0 (0.00)	0 (0.00)	0 (0.00)	0 (0.00)	0 (0.00)	-	0 (0.00)
R	30 (78.95)	3 (17.65)	0 (0.00)	8 (61.54)	0 (0.00)	-	1 (33.33)
Itraconazole							
S	33 (86.84)	16 (94.12)	16 (100.0)	11 (84.62)	10 (100.0)	-	3 (100.0)
SDD	-	-	-	-	-	-	-
I	0 (0.00)	0 (0.00)	0 (0.00)	0 (0.00)	0 (0.00)	-	0 (0.00)
R	5 (13.16)	1 (5.88)	0 (0.00)	2 (15.38)	0 (0.00)	-	0 (0.00)
Amphotericin B							
S	37 (97.37)	17 (100.0)	15 (93.75)	13 (100.0)	10 (100.0)	7 (87.8)	3 (100.0)
SDD	-	-	-	-	-	-	-
I	0 (0.00)	0 (0.00)	0 (0.00)	0 (0.00)	0 (0.00)	0	0 (0.00)
R	1 (2.63)	0 (0.00)	1 (6.25)	0 (0.00)	0 (0.00)	1 (12.5)	0 (0.00)
Caspofungin							
S	35 (92.11)	17 (100.0)	16 (100.0)	8 (61.54)	10 (100.0)	4 (50.00)	1 (33.33)
SDD	-	-	-	-	-	-	-
I	1 (2.63)	0 (0.00)	0 (0.00)	4 (30.77)	0 (0.00)	0 (0.00)	2 (66.67)
R	2 (5.26)	0 (0.00)	0 (0.00)	1 (7.69)	0 (0.00)	4 (50.00)	0 (0.00)
Anidulafungin							
S	33 (86.84)	17 (100.0)	16 (100.0)	13 (100.0)	10 (100.0)	8 (100.0)	3 (100.0)
SDD	-	-	-	-	-	-	-
I	0 (0.00)	0 (0.00)	0 (0.00)	0 (0.00)	0 (0.00)	0 (0.00)	0 (0.00)
R	5 (13.16)	0 (0.00)	0 (0.00)	0 (0.00)	0 (0.00)	0 (0.00)	0 (0.00)
Micafungin							
S	35 (92.11)	17 (100.0)	16 (100.0)	13 (100.0)	9 (90.00)	8 (100.0)	3 (100.0)
SDD	-	-	-	-	-	-	-
I	0 (0.00)	0 (0.00)	0 (0.00)	0 (0.00)	0 (0.00)	0 (0.00)	0 (0.00)
R	3 (7.89)	0	0 (0.00)	0 (0.00)	1 (10.00)	0 (0.00)	0 (0.00)

All *C. albicans* isolates were fully susceptible to amphotericin B, caspofungin, anidulafungin, and micafungin. In contrast, *C. parapsilosis* was partially resistant to fluconazole (4, 25%) and amphotericin B (1, 6.25%), along with complete susceptibility to other antifungal agents. *N. glabrata* displayed a markedly high resistance to voriconazole (11, 84.62%) and posaconazole (8, 61.54%), followed by fluconazole (3, 23.08%), itraconazole (2, 15.38%), and caspofungin (1, 7.69%). Notably, 4 (30.77%) of the isolates were intermediately susceptible to caspofungin, and 10 (76.92%) exhibited dose-dependent susceptibility to fluconazole. Additionally, *C. orthopsilosis* demonstrated resistance to fluconazole, voriconazole, and micafungin in only one isolate (10%) each.

Among *C. auris* isolates, fluconazole resistance was the highest (7, 87.5%), followed by caspofungin (4, 50%). Furthermore, amphotericin B resistance was detected in only one isolate, whereas all isolates were susceptible to anidulafungin and micafungin. Due to the absence of established MIC breakpoints (in µg/mL) for voriconazole, posaconazole, and itraconazole in *C. auris*, the geometric mean MIC values for these drugs were calculated as 2.69, 0.60, and 1.22 µg/mL, respectively. The geometric mean MIC values of antifungal agents against less common *Candida* isolates, including *C. jadinii*, *K. ohmeri*, *Candida* *metapsilosis*, *C. fabianii*, and *C. haemulonii*, have been summarized in Table 3.

**Table 3:** Geometric mean MIC values of antifungal agents against less common Candida isolates. Values are expressed as the geometric mean of the minimum inhibitory concentration (MIC).

Antifungal medication	Name of the organisms				
	*Cyberlindnera jadinii* (*n* = 5) (Mean MIC)	*Kodamaea ohmeri* (*n* = 5) (Mean MIC)	*Candida metapsilosis* (*n* = 1) (MIC)	*Cyberlindnera fabianii* (*n* = 1) (MIC)	*Candida haemulonii* (*n* = 1) (MIC)
Fluconazole	3.482	8	4	1	256
Voriconazole	0.16	0.079	0.06	0.015	8
Posaconazole	0.5	0.06	0.06	0.25	1
Itraconazole	0.247	0.138	0.25	0.25	16
Amphotericin B	0.5	0.378	0.5	0.25	1
Caspofungin	0.079	0.751	0.25	0.015	0.12
Anidulafungin	0.041	0.321	0.5	0.015	0.12
Micafungin	0.03	0.375	0.5	0.06	0.06
5-flucytosine	0.06	0.06	0.06	0.06	0.06

## Discussion

In recent years, *Candida* species have been emerging pathogens identified as a leading cause of BSIs and are associated with significant mortality among patients with sepsis. In the present study, a prevalence of 0.73% was observed, which is similar to the multicenter study by Ghrenassia et al. [16]. At present, there has been a significant epidemiological shift observed in India from C. albicans to NAC species as the predominant cause of candidemia [17,18]. A similar trend was also elucidated in our study. This increased isolation of NAC could be attributed to the exacerbated irrational overuse of fluconazole for the treatment of all fungal infections, which has resulted in the emergence of fluconazole-resistant NAC strains. Additionally, improved species identification and detection of NAC may have contributed to the same [17].

The present study identified NAC candidemia to be more prevalent in the adult age group (54, 53.4%). In contrast, prior research has reported a higher incidence of candidemia in the pediatric age group, particularly among neonates [4,17]. We also observed a slightly higher prevalence in males (68, 57.63%) than in females (50, 42.37%), which was concurrent with the findings of Zeng et al. [19]. Furthermore, Zeng et al. noted that candidemia was more common in patients in the general ward than in ICUs [19], which was similarly observed in our study (69.5% vs. 30.5%). This could be attributed to the higher number of blood culture samples received from the patients in the ward.

The distribution and antifungal susceptibility of *Candida* species may vary widely across different geographical regions and healthcare settings. In the present study, *C. tropicalis* was the most frequently isolated species, accounting for 32.20% of the cases, followed by *C. albicans*, *C. parapsilosis*, *N. glabrata*, *C. orthopsilosis*, and *C. auris*. The predominance of *C. tropicalis* as the most common isolate in our candidemia cases is consistent with multiple reports from India [1,5]. In contrast, Babu et al. reported other species, such as *C. parapsilosis* or *C. albicans*, as the most common species in candidemia cases [17]. Our results revealed that *C. auris*, an emerging multidrug-resistant pathogen, was isolated in 6.78% (8/118) of the cases. In contrast, a recent study from India reported a lower incidence (0.97%) of *C. auris* in candidemia [20]. In addition to these predominant *Candida* species, the present study also identified the presence of several less common or rare *Candida* species, including *Cyberlindnera jadinii*, *K. ohmeri*, *C. metapsilosis*, *C. fabianii*, and *C. haemulonii*. These species have also been reported in previous studies as causative agents of BSIs or invasive candidiasis [21,22].

Our investigation provided valuable insights into the antifungal susceptibility profiles of commonly isolated *Candida* species. Among the azole drugs, posaconazole is generally considered to have good fungicidal activity and lower resistance rates against most *Candida* species [23]. Unfortunately, the present study observed a higher resistance to posaconazole (30, 78.95%) in *C. tropicalis*, which raises concerns over the treatment of candidemia and highlights the importance of AFST to identify the resistant isolates. In contrast, Navarathinam et al. reported lower resistance to posaconazole in *C. tropicalis* isolates [23]. This study also noted relatively less resistance towards fluconazole, voriconazole, and itraconazole in our isolates. Over the years, a rising trend in fluconazole resistance in *C. tropicalis* has been reported [24]. In comparison, our study indicated a lower resistance to fluconazole in *C. tropicalis* (18.42%), thereby warranting the need for further research on fluconazole resistance. Furthermore, the low resistance rates observed for amphotericin B and echinocandins in this study support the continued suitability of these agents as first-line treatment options for *C. tropicalis* candidemia.

Although *C. albicans* remains the leading cause of BSIs, it has exhibited relatively low antifungal resistance across all classes of antifungal drugs compared to other *Candida* species. Similarly, *C. albicans* isolates in the present study demonstrated higher susceptibility to all tested antifungals, except posaconazole. *C. parapsilosis* has historically been considered susceptible to azoles; however, fluconazole resistance has recently been increasing in some countries [25,26]. The present study reported higher resistance of 25% to fluconazole in *C. parapsilosis* isolates, which correlates with the findings of Arastehfar et al. [27]. In our study, the *N. glabrata* strain exhibited high resistance to azole antifungals, particularly voriconazole (84.62%) and posaconazole (8, 61.54%), along with good susceptibility to echinocandins and amphotericin B. In contrast, Castanheira et al. reported lower resistance to azole antifungals in *N. glabrata* [28]. The higher azole resistance observed in our isolates could be due to several factors, including the fact that *N. glabrata* is a haploid organism and resistance can be conferred by a single mutation that facilitates the development of antifungal drug resistance [12].

*C. auris* is an emerging multidrug-resistant pathogen that poses a significant threat to public health due to its ability to rapidly develop resistance to all three major classes of antifungal drugs, thus making the treatment challenging. In our study, *C. auris* was reported in 6.78% of cases, whereas Shastri et al. reported a higher incidence of *C. auris* in candidemia [29]. Moreover, 87.5% of our *C. auris* isolates were resistant to fluconazole. This finding is consistent with previous studies by Biagi et al. [30] and Shastri et al., which reported fluconazole resistance rates of approximately 90% and 97%, respectively [29]. In the present study, *C. auris* isolates demonstrated 100% susceptibility to anidulafungin and micafungin, thereby suggesting that these echinocandins remain highly effective treatment options. This was also concurrent with the findings of Biagi et al. [30]. In our study, 12.5% amphotericin B resistance in *C. auris* isolates was observed, which is lower than the other studies [13,31]. Furthermore, our results demonstrated 50% resistance to caspofungin in *C. auris* isolates, which indicated the need for cautious use of this drug in treatment.

In addition to the predominant Candida species, our study identified the presence of several less common or rare Candida species, including *C. jadinii*, *K. ohmeri*, *C. metapsilosis*, *C. fabianii*, and *C. haemulonii*. Prior studies have also reported these species in cases of BSIs or invasive candidiasis [32]. Given the limited availability of established clinical breakpoints or epidemiological cutoff values for these species, the present study determined the MICs of antifungal agents against these isolates and expressed the results as geometric mean values (Table 3). Consequently, these preliminary observations highlighted the emerging clinical relevance of these uncommon species and underscore the need for continuous surveillance and susceptibility profiling. As this is an initial observation, further investigations are underway to better understand the antifungal resistance patterns and potential clinical implications associated with these pathogens.

Overall, our data underscores the importance of routine, species-specific AFST, given the significant interspecific differences observed in antifungal response. The susceptibility trends observed in this study have direct implications for antifungal stewardship and highlight the urgent need to establish or strengthen antifungal stewardship programs. Furthermore, early identification and prompt species-specific treatment can significantly improve patient outcomes. Continuous surveillance is essential, as the rapidly changing resistance profiles demand frequent monitoring. Our study has certain limitations: the data were derived from a single center, which may not fully represent the epidemiological trends of candidemia across different regions of India. Additionally, the analysis was based on a relatively small number of isolates collected from patients, which may not represent all hospital-acquired candidemia cases. Moreover, due to the retrospective nature of this study, detailed clinical data for each patient could not be assessed. Also, the study is limited by the lack of clinical outcome data and the absence of molecular resistance analysis.

## Conclusions

The increasing prevalence of NAC species and the rising resistance to commonly used antifungals, particularly azoles, underscore the importance of timely diagnosis, species-level identification, AFST, and prompt, species-specific antifungal therapy for candidemia. The observed high antifungal drug resistance to posaconazole (particularly in *C. tropicalis*) and fluconazole (in *C. auris* and *C. parapsilosis*) across several Candida isolates highlights the importance of routine antifungal susceptibility testing for BSI isolates. Echinocandins are generally effective against most Candida species; however, they should be used cautiously in patients with *C. auris* BSI, as *C. auris* isolates may exhibit multidrug resistance, including resistance to echinocandins. Future research is recommended to validate these resistance trends in a multicenter setting and better define the clinical implications of MIC values for rare *Candida* species.
